# Analysis of different standards for life support technology in manned submersible

**DOI:** 10.3389/fphys.2024.1304250

**Published:** 2024-10-10

**Authors:** Lei Jiang, Cong Ye, Hongxia Li, Yao Wang, Nian Jiang

**Affiliations:** ^1^ China Ship Scientific Research Center, Wuxi, China; ^2^ School of Business, Jiang Nan University, Wuxi, Jiangsu, China; ^3^ Taihu Laboratory of Deepsea Technological Science, Wuxi, China; ^4^ State Key Laboratory of Deep-sea Manned Vehicles, Wuxi, China; ^5^ Wuxi Institute of Technology, Wuxi, China; ^6^ Yantai Graduate School, Harbin Engineering University, Yantai, China

**Keywords:** ISO standard, life support technology, manned submersible, analysis, data confirm

## Abstract

Life support technology is one of the most critical technologies in manned submersibles, acting for the pilots of the submersible and thus directly affecting its underwater safety. Due to its importance, many countries and international organizations have proposed standards and specifications for life support technology. This paper presents an overview of the life support technology of manned submersibles, comparing the standards proposed for it. Furthermore, it analyzes the specific data of oxygen supply and carbon dioxide absorption, both of which are critical aspects of life support technology in each standard, and identifies the data that is widely acknowledged and recognized as the basis for establishing ISO standard 22252:2020). Finally, ISO standard data such as oxygen and carbon dioxide concentration are further confirmed by the environmental parameters of manned submersibles such as the *Fendouzhe*.

## 1 Introduction

It has been more than 60 years since the first self-propelled manned submersible, the *Diving Saucer*, was launched in 1959. Throughout history, humans have relied on the most advanced scientific and technological achievements available to understand, develop, and protect the oceans. Manned submersibles are at the forefront of these advanced scientific and technological developments. These vehicles are capable of transporting scientists, engineers, and technical personnel, along with electronic devices and special equipment, to various deep-sea complex environments rapidly and accurately. They facilitate efficient exploration, scientific investigation, and seabed operations. Manned submersibles are also important technical means for the peaceful development and utilization of deep-sea resources. To a certain extent, they represent a nation’s ability to explore and develop marine resources and safeguard maritime rights and interests and the level of science and technology. As countries around the world intensify their research into ocean exploration, especially in the deep sea, manned submersible projects are being increasingly developed. Representative manned submersibles are the *Alvin* (Kohnen, 2009) of the United States, *Nautile* (Lévêque and Drogou, 2006) of France, *Mir I* and *Mir II* (Sagalevitch, 2009) of the former Soviet Union, *Shinkai 6500* (Morihana et al., 1990) of Japan, and China’s *Jiaolong*, *Deep Sea Warrior*, and *Fendouzhe* (Wu et al., 2002). These submersibles have been integrated with a variety of technologies for life support, communication and navigation, electronic information, and mechanics and hydraulics. Life support technology is a component of submersible technology which strives to create a normal or even comfortable working environment for occupants, such as providing the precise oxygen needed for them to breathe and efficiently removing the carbon dioxide they exhale. However, although submersibles all use life support technology, there are different methods and technical parameters due to different industrial systems, and this does not favor the international technical exchange for submersibles. In the long run, the design, testing, and use of submersible will trend toward international cooperation. In order to enhance the universality of submersible technology, it is necessary to conduct research into and formulate submersible standards. This is of great significance for standardizing submersible design concepts and setting the direction of submersible development. Therefore, it is significant to research and formulate national or international standards for manned submersible technology (Ma et al., 2017).

## 2 Overview of standards for manned submersibles

As research on manned submersibles becomes increasingly in-depth, standards must be quickly updated for meticulous discipline division and high standards. Many organizations or institutions have proposed standards or specifications for manned submersibles, such as the American Bureau of Shipping (ABS), Lloyd’s Register of Shipping (LR), and the China Bureau of Shipping Inspection. In addition, the International Maritime Organization (IMO), an international body, has also issued mandatory regulations on manned submersibles. For the 21^st^ century, the International Organization for Standardization (ISO) began researching and developing manned submersible standards.

In February 2015, the ISO Marine Technology Sub-Committee (ISO/TC8/SC13) officially established the first working group under its jurisdiction: a submersible working group, number ISO/TC8/SC13/WG1, which is fully responsible for formulating and revising all ISO standards pertaining to submersibles. Its objective is to develop standards on test methods, operating procedures, equipment and facilities, technical means, and other aspects related to marine observation and protection and resource exploration*.* Furthermore, the working group aims to provide unified standards and operating procedures for global marine investigation and observation and promote the establishment of underwater observation systems, the utilization of marine resources, and the development of new marine equipment manufacturing industry (Feng et al., 2020). Table 1 shows some of the latest standards or specifications for manned submersibles commonly used in the industry. It shows that the publishers of these standards consist of non-government organizations such as classification societies, international organizations such as IMO and ISO, and the standardization committees of certain countries.

**TABLE 1:** Standards related to manned submersibles.

No.	Standard name	Standard number or code	Publisher	Publish time	Level of standard	Standard effectiveness
1	Guidelines for the Design, Construction and Operation of Passenger Submersible Craft	MSC/Circ. 981	International Maritime Organization (IMO)	2001	International convention	Compulsory
2	Submersibles--Hydrostatic Pressure Test --Pressure Hull and Buoyancy Materials	ISO 21173	International Organization for Standardization (ISO)	2019	International standard	Recommended
3	Manned Submersibles--Breathing Air Supply and CO_2_ Adsorption Systems—Performance Requirements and Recommendations	ISO 22252	International Organization for Standardization (ISO)	2020	International standard	Recommended
4	Oxygen Supplying and Carbon Dioxide Absorbing for Human Occupied Vehicles	GB/T35371-2017	Standardization Administration of China (SAC)	2017	National standard	Recommended
5	Specification for Classification of Diving Systems and Submersibles	—	China Classification Society (CCS)	2018	Industry standards	Recommended
6	Rules for Building and Classing Underwater Vehicles, Systems and Hyperbaric Facilities	—	American Bureau of Shipping (ABS)	2018	Industry standards	Recommended
7	Rules and Regulations for the Construction and Classification of Submersibles and Diving Systems	—	Lloyd’s Register of Shipping (LR)	2017	Industry standards	Recommended

An in-depth study of the above standards or specifications shows that the standards and proposals of classification societies are mostly tailored to the specific conditions of the country in which they are located, consolidating all the technologies involved in manned submersibles into a single set of agreed-upon specifications. As an international convention with mandatory effect, IMO standards primarily focus on aspects such as personnel and equipment safety. However, they do not provide extensive regulations on the specific technical aspects and design details of manned submersibles. ISO standards tend to divide manned submersible technology into specific areas, such as hydrostatic pressure test, oxygen supply, and carbon dioxide absorption. They then propose specific and detailed standards for each of these areas.

From the perspective of the users of standards, the ISO’s practices of standard preparation and publication tend to be developed for specific technologies or individual devices, followed by a series of specific standards for large-scale equipment or complex technologies.

Multiple countries’ technical standards or specifications regarding manned submersibles are listed in items 4–7 of Table 1, and these play a significant role in the design process of manned submersibles.

However, these standards have some limitations in terms of their scope of application and recognition. At present, the widely used standards for manned submersibles are formulated by the standards committee or classification society of each country. These standards, when formulated, primarily consider the technical capacities and levels of the respective countries without necessarily taking into account the international applicability of the standard. Due to economic and even political factors, these standards may even set some exclusionary provisions, thereby affecting the standard’s global recognition.

To some extent, ISO standards compensate for the shortcomings of national standards. Because they require global participation and assent, the development process of ISO standards determines their wide applicability compared to national standards. The need for ISO standards for manned submersibles has thus become increasingly urgent.

## 3 Comparison of life support technology of manned submersibles in different standards

Life support technology is commonly used in the manned closed compartments of submarines, submersibles, manned spacecraft, and other such vehicles. Since these vehicles operate in places such as water or a vacuum where human beings cannot directly survive or breathe, their crews or passengers cannot obtain fresh air from the external environment. Therefore, the vehicle must provide a self-contained breathing and survival environment (Jiang et al., 2019).

Life support technology includes elements such as air conditioning and ventilation, a breathing gas supply, air purification, and atmospheric environmental monitoring. In the specific parameters of these technologies, abnormal oxygen and carbon dioxide levels will threaten the life safety of passengers; these two gases thus have specific criteria and are the focus of standards.

The ISO 22252 standard, prepared by the ISO/TC8/SC13/WG1 working group, focuses on breathing gas supply and carbon dioxide absorption. Therefore, during the standard’s development process, the working group conducted extensive comparative analysis on four specific parameters—oxygen supply, oxygen concentration in the manned cabin, carbon dioxide removal capacity, and carbon dioxide concentration in the manned cabin—in relation to various national standards.

The comparison of the oxygen supply data is shown in Table 2.

**TABLE 2:** Comparison of oxygen supply requirements for manned submersibles in different standards.

Publisher	Standard name	Standard number or code	Content	Chapter number	Year published
IMO	Guidelines for the Design, Construction and Operation of Passenger Submersible Craft	MSC/Circ. 981	28.3 *l/h*·*per person* (International Maritime Organization, 2001)	Section 2.4.4.4	2001
ABS	Rules for Building and Classing Underwater Vehicles, Systems and Hyperbaric Facilities	—	0.038 *kg/h·per person* at 1 *atm* (American Bureau of Shipping, 2018)	Section 8.5.5	2018
LR	Rules and Regulations for the Construction and Classification of Submersibles and Diving Systems	—	≥30 *l/h per*·*person* (measured at 20 °C) (Lloyd’s Register of Shipping, 2004)	Pt5, Ch4 Section 5.1.4	2017
CCS	Specification for Classification of Diving Systems and Submersibles	—	≥25 *l/h*·*per person* at 1 *atm* (China Classification Society, 2018)	Section 8.3.1.2	2018
SAC	Oxygen Supplying and Carbon Dioxide Absorbing for Human Occupied Vehicles	GB/T35371-2017	≥25 *l/h*·*per person* at 1 *atm* (Standardization Administration of China, 2017)	Section 5.2.1	2017

All national standards have relatively clear descriptions of oxygen supply, but there are differences in the specific value or description method. Table 2 shows that, except for the IMO, all standards refer to what environmental pressure or ambient temperature the oxygen supply should correspond. Since the volume of the gas is significantly affected by ambient pressure and temperature, the temperature and pressure in the manned cabin of the submersible will change significantly when it is underwater (Jiang and Hou, 2011). Therefore, to be rigorous, ISO 22252 expands the description of ambient temperature and pressure. In addition, through comparison, it can be found that the specific value of oxygen supply of each standard is very different. The LR standard is the highest, reaching 30 L/hper person. The values in ABS and IMO are basically the same, reaching the second highest level. CCS and China’s national standard stipulate 25 L/h per person, which is significantly lower than in other specifications.

Considering the universality of the standard and the compulsory nature of IMO regulations, ISO 22252 takes more direction from IMO and ABS regulations in determining this value.

The second parameter is the comparison of oxygen concentration indicators (Table 3).

**TABLE 3:** Comparison of oxygen concentration requirements for manned submersibles in different standards.

Publisher	Standard name	Standard number or code	Content	Chapter number	Year published
IMO	Guidelines for the Design, Construction and Operation of Passenger Submersible Craft	MSC/Circ. 981	18%–23% by volume	Section 2.4.4.2	2001
ABS	Rules for Building and Classing Underwater Vehicles, Systems and Hyperbaric Facilities	—	1 *atm* chambers/systems: 18%–23% by volume	Section 11.35.1	2018
LR	Rules and Regulations for the Construction and Classification of Submersibles and Diving Systems	—	1 *atm* submersibles, long term exposure, 150 *mmHg* (0.2*bar*) - 0.5 *barg*	Pt5, Ch4 Section 5.2.1	2017
CCS	Specification for Classification of Diving Systems and Submersibles	—	18%–23% by volume	Section 8.1.2.2	2018
SAC	Oxygen Supplying and Carbon Dioxide Absorbing for Human Occupied Vehicles	GB/T35371-2017	18%∼23%	Section 5.1.1	2017

These standards are more consistent, except for LR, which specifies requirements in the form of oxygen partial pressure value, while the rest specify requirements in the form of volume percentage. Both the partial pressure value and the volume percentage can be used to characterize the gas concentration. The difference is that the partial pressure value is often used when environmental pressure exceeds 1 atm, and the volume percentage is generally exactly or approximately 1 atm. Therefore, the ISO 22252 standard chooses the percentage of volume as the unit to characterize oxygen concentration.

The third parameter is the comparison of carbon dioxide removal capacity (Table 4).

**TABLE 4:** Comparison of carbon dioxide removal capacity requirements for manned submersibles in different standards.

Publisher	Standard Name	Standard number or code	Content	Chapter number	Year published
IMO	Guidelines for the Design, Construction and Operation of Passenger Submersible Craft	MSC/Circ. 981	≥0.053 *kg/h per person*	Section 2.4.4.4	2001
ABS	Rules for Building and Classing Underwater Vehicles, Systems and Hyperbaric Facilities	—	≥0.0523 *kg/h per person* at 1 *atm*	Section 8.5.5	2018
LR	Rules and Regulations for the Construction and Classification of Submersibles and Diving Systems	—	≥17.5*L/h per person* (measured at 20°C)	Pt5, Ch4 Section 7.2.1	2017
CCS	Specification for Classification of Diving Systems and Submersibles	—	≥22 *l/h·per person* at 1 *atm*	Section 8.4.2.1	2018
SAC	Oxygen Supplying and Carbon Dioxide Absorbing for Human Occupied Vehicles	GB/T35371-2017	≥22 *l/h·per person* at 1 *atm*	Section 6.2.1	2017

The specific value of each standard for carbon dioxide removal capacity is different. The standard values of IMO and ABS are basically the same, and they are also the most demanding in each standard, while the standard requirements of LR are the lowest; the rest are basically the same. Considering that carbon dioxide concentration can cause serious harm to the human body, it is necessary from a safety perspective to use high-standard requirements. Therefore, in setting this value, ISO 22252 has taken more direction from the IMO and ABS provisions, while adding descriptions of the environmental pressure and ambient temperature in the standard.

The fourth parameter is the comparison of carbon dioxide concentration requirements (Table 5).

**TABLE 5:** Comparison of carbon dioxide concentration requirements for manned submersibles in different standards.

Publisher	Standard name	Standard number or code	Content	Chapter number	Year published
IMO	Guidelines for the Design, Construction and Operation of Passenger Submersible Craft	MSC/Circ. 981	≤0.5% (normal) ≤1% (emergency) by volume	Section 2.4.4.2	2001
ABS	Rules for Building and Classing Underwater Vehicles, Systems and Hyperbaric Facilities	—	≤0.5% by volume (a CO_2_ mass of 0.00989 *kg/m* ^*3*^ at 1 *atm* and 0°C)or 0.005 *ata*	Section 8.9.1	2018
LR	Rules and Regulations for the Construction and Classification of Submersibles and Diving Systems	—	≤5.0 *mbar* (normal) ≤ 10 *mbar* (at the end of the emergency life support period)	Pt5, Ch4 Section 7.3.1	2017
CCS	Specification For Classification of Diving Systems and Submersibles	—	≤0.5 kpa (normal) ≤1 kpa (emergency)	Section 8.4.1.1 Section 8.5.4.1	2018
SAC	Oxygen Supplying and Carbon Dioxide Absorbing for Human Occupied Vehicles	GB/T35371-2017	≤0.5% (normal) ≤1% (spare time) ≤1.5% (emergency)	Section 6.2.1	2017

The standards here vary greatly. Some use partial pressure value as the unit for carbon dioxide concentration, while others use volume percentage. Different standards have different criteria for distinguishing the working states of the submersible. Apart from ABS, all other standards classify the working state of submersibles into normal state and emergency states, and then impose different requirements for each state. The specific working state values of each standard are not the same.

Regarding the first difference, ISO 22252 specifies the use of volume percentage as the unit for gas concentration after specifying requirements for environmental temperature and pressure. In the second difference, due to the limited energy reserve and cabin space (Liu et al., 2008), existing manned submersibles divide their operational states into normal and emergency. The normal state prioritizes regular operations with high performance requirements, while the emergency state prioritizes survival with lower performance requirements. If only conventional metrics of operational time are used as requirements for carbon dioxide concentration, the energy consumption and volume of carbon dioxide removal equipment will inevitably increase, which is a relatively large burden for a submersible. Therefore, ISO 22252 also distinguishes the working state of the submersible. For the third difference, ISO 22252 chooses a stricter concentration requirement as a reference for safety.

After the above comparison, analysis and selection, the specific technical parameters of oxygen supply, oxygen concentration, carbon dioxide removal capacity, and carbon dioxide concentration requirements in ISO 22252 are formed (Table 6).

**TABLE 6:** Some specific technical parameters of ISO 22252 (International Organization for Standardization, 2020).

Oxygen supply (°C)	Oxygen concentration	CO_2_ removal ability (°C)	CO_2_ concentration
28.3 *l/h*·*per person* at 1 *atm* and 20	18%–23% by volume	≥0.053 *kg/h·per person* at 1 *atm* and 20	≤0.5% by volume (normal and reserve time) ≤1% by volume (emergency time)

## 4 Confirmation of specific technical parameters in ISO 22252

To verify the feasibility and effectiveness of the specific technical parameters proposed in Table 6, it is necessary to compare them with actual data for existing manned submersibles. This will help determine whether the technical parameters in the standard can cover the indicators of existing submersibles to help evaluate whether they can guide the design of new manned submersibles. We thus select three types of manned submersibles with different missions and obtain their relevant data for analysis.

The three kinds of submersibles are the *Fendouzhe*, designed and manufactured in China for scientific research, a passenger-carrying submersible designed and manufactured in China for tourism, and the *LR7*, designed and manufactured in the United Kingdom for rescue missions.

**FIGURE 1:**
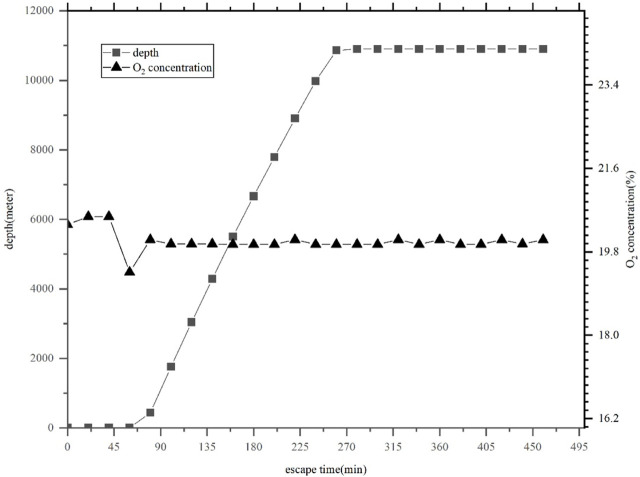
Oxygen concentration curve in the manned cabin of the *Fendouzhe* during a dive.

**FIGURE 2:**
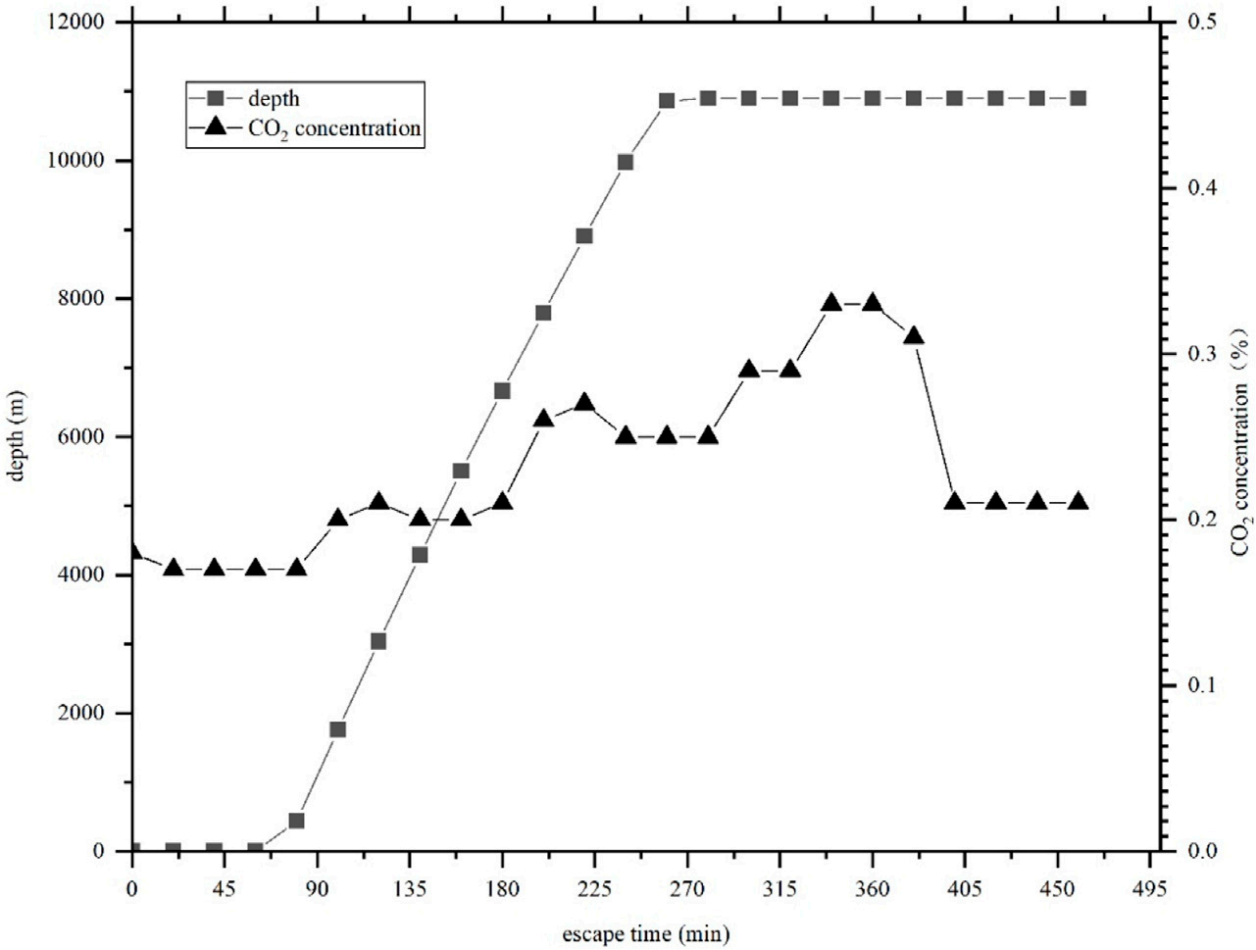
Carbon dioxide concentration curve in the manned cabin of the *Fendouzhe* during a dive.

Figures 1 and 2 show the curve of oxygen and carbon dioxide concentrations in the manned cabin of the *Fendouzhe* when it dives to approximately 10,900 m below sea level.

The *Fendouzhe* is designed with an oxygen supply rate and carbon dioxide removal capacity of 25 L/h and 22 L/h per person, respectively. Figures 1 and 2 show that during a descent of approximately 500 min, the oxygen concentration in the manned cabin is maintained between 19% and 22%, and at a long time below the normal atmospheric oxygen concentration of about 20.9%. The concentration of carbon dioxide is kept below 0.4%.

According to the data, the oxygen and carbon dioxide concentrations stipulated in ISO 22252 standard can be applied to the *Fendouzhe*. However, the standard requirements for oxygen supply and carbon dioxide removal capacity are slightly higher than the design targets of this submersible. Therefore, if the *Fendouzhe* is designed according to ISO 22252, the oxygen concentration in the manned cabin will be increased to be closer to normal atmospheric oxygen concentration, and the carbon dioxide concentration will be lower; both are more comfortable and safer for the submersible’s passengers. Therefore, when considering safety, using this ISO standard as a guide for the design of a research submersible without affecting its overall performance or causing a significant impact can be helpful in enhancing its safety.

**FIGURE 3:**
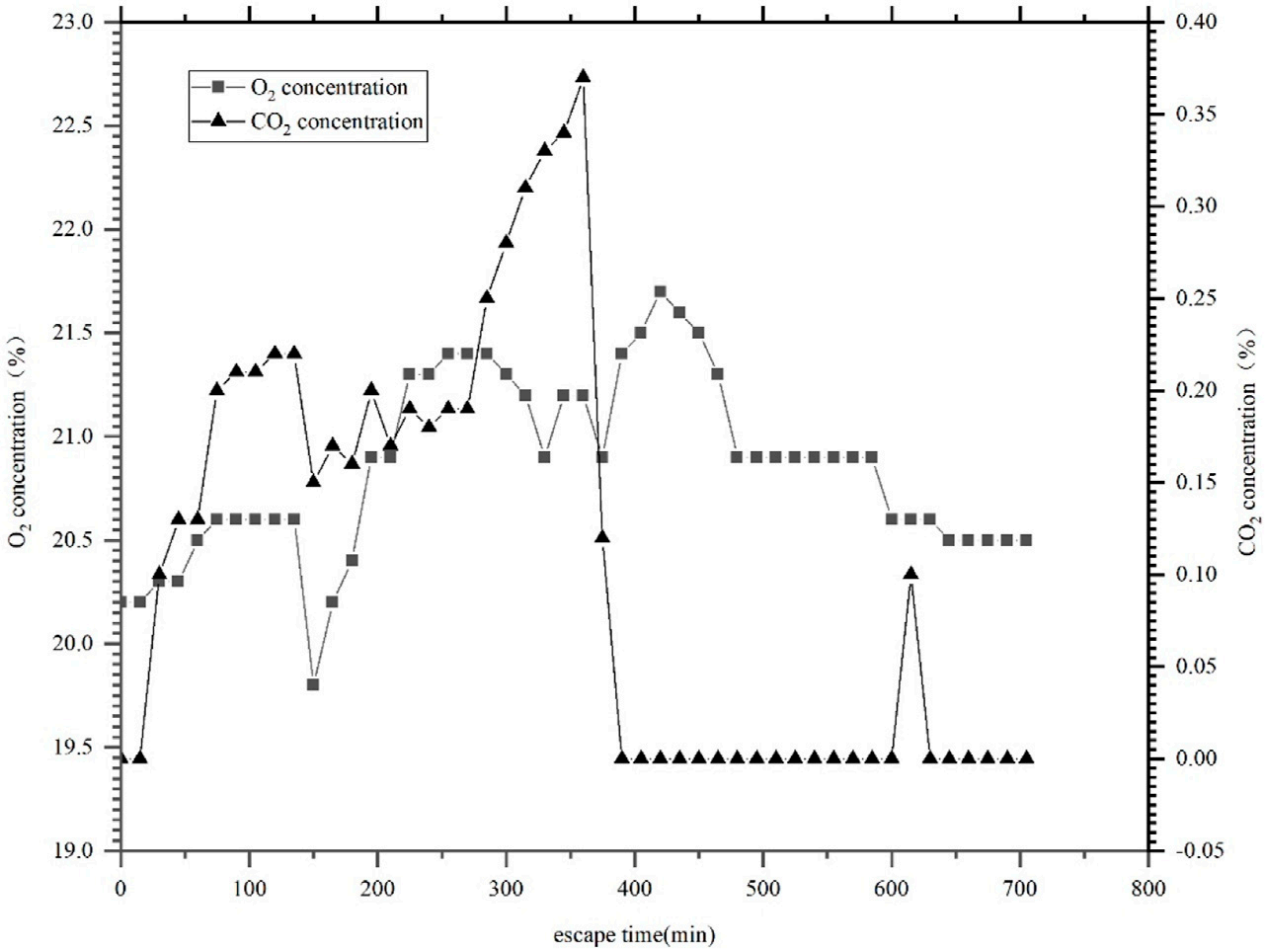
Oxygen and carbon dioxide concentration curve in the manned cabin of the passenger submersible during a dive.

Figure 3 shows the curve of oxygen and carbon dioxide concentrations in the manned cabin of a tourist passenger submersible. The oxygen supply and CO_2_ removal capacity of the submersible are designed according to 30 and 22 L/hper person respectively.

Figure 3 shows that the dive lasted for approximately 12 h, during which the oxygen concentration fluctuated between 19.8% and 21.8%, the carbon dioxide concentration remained below 0.36%, and the oxygen concentration exceeded the normal atmospheric oxygen concentration (about 20.9%) for approximately 6 h.

From Figure 3 and related technical data, it can be seen that the provisions of ISO 22252 on oxygen and carbon dioxide concentrations can apply to the submersible. However, the requirements of oxygen supply in the standard are lower than the design requirements of the submersible, while the requirements of carbon dioxide removal capacity are stricter than the design indicators of the submersible. Therefore, if the design of the tourism manned submersible is guided by ISO 22252, the oxygen concentration in the manned cabin will be closer to conventional atmospheric oxygen concentration, and the carbon dioxide concentration will be lower than the current situation, which will be helpful for improving the submersible’s safety.

**FIGURE 4:**
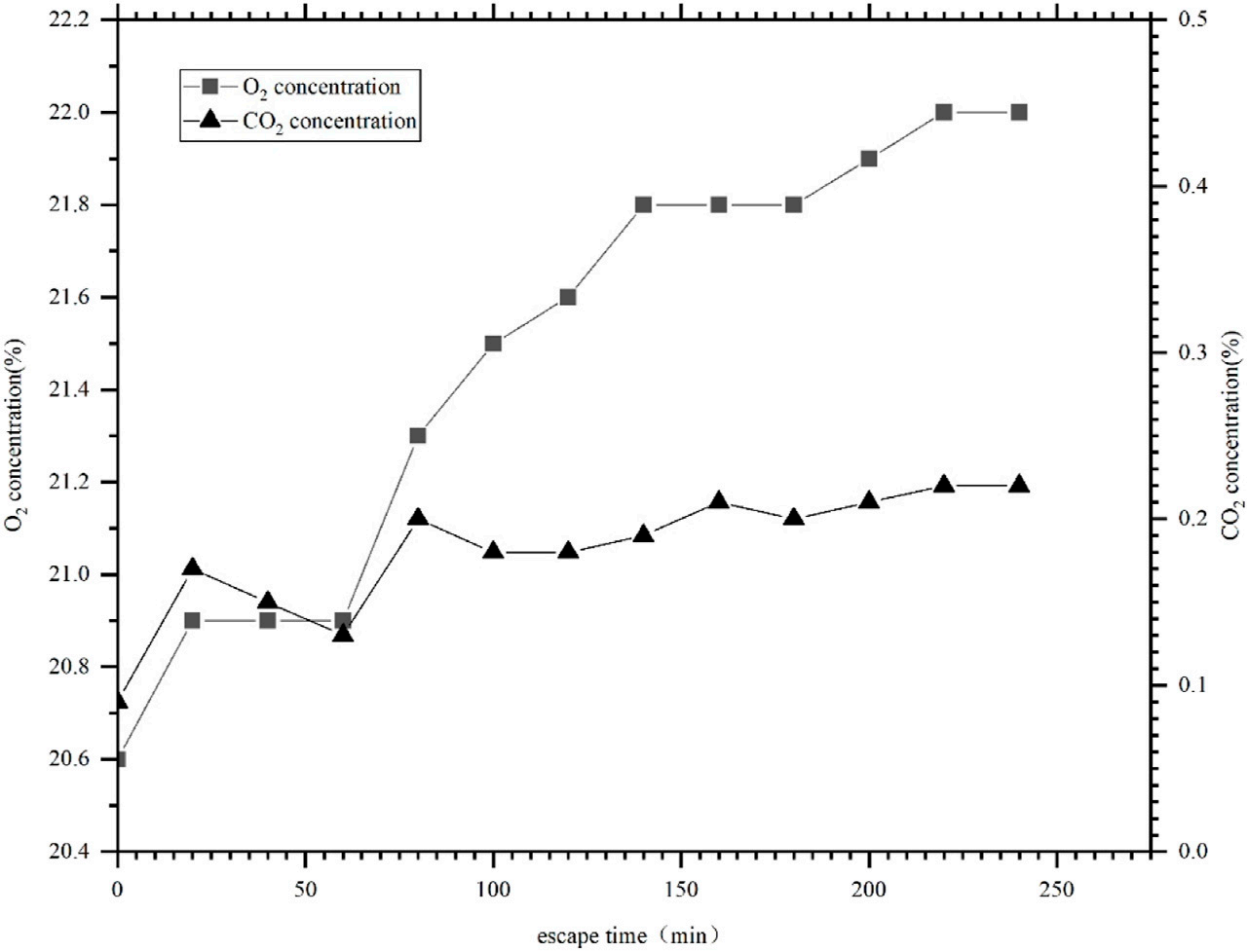
Oxygen concentration and carbon dioxide concentration curve in the manned cabin of the *LR7* rescue submersible.

Figure 4 shows the curve of oxygen concentration and carbon dioxide concentration in the manned cabin of the *LR7* rescue submersible.

The submersible’s oxygen supply and carbon dioxide removal capacity are designed to be to 30 L and 17.5 L/hper person respectively. Figure 4 shows that the dive lasted for approximately 4 h, during which the oxygen concentration fluctuated between 20.6% and 22% while the carbon dioxide concentration remained below 0.23%. During the whole process, the oxygen concentration was significantly higher, and a rising trend was obvious after 60 min. This is directly related to the limited cabin capacity of the submersible and the design of oxygen supply capacity based on 30 L/hper person.

The graph and related technical data show that the oxygen and carbon dioxide concentrations stipulated in ISO 22252 can apply to this submersible. However, the requirements of oxygen supply in ISO 22252 are lower than that of the submersible, and the requirements of carbon dioxide removal capacity are stricter than the design indicators of the submersible. If the submersible is designed according to ISO 22252, the oxygen concentration in the manned cabin will be more similar to the conventional atmospheric oxygen concentration, and the carbon dioxide concentration will be lower than the current situation. Therefore, the use of ISO 22252 to guide the design of submersibles is conducive to improving their safety.

## 5 Conclusion

Many countries and organizations have introduced standards or specifications for manned submersibles, but their specific descriptions, requirements, scope, and specificity vary. This paper introduces common standards or specifications for guiding life support technology in manned submersibles. With applicability as the starting point, this paper compares, analyzes, and selects the specific technical data and requirements of these standards or specifications from the perspective of writing ISO standards, ultimately forming the specific technical parameters of ISO 22252. Finally, this paper uses the environmental parameters of manned submersible cabins with different mission objectives to evaluate the specific technical parameters of ISO 22252, confirming their ability to cover existing manned submersibles and guide future submersible design.

## Data Availability

The original contributions presented in the study are included in the article/supplementary material; further inquiries can be directed to the corresponding author.
